# A Multi-Hop Clustering Mechanism for Scalable IoT Networks

**DOI:** 10.3390/s18040961

**Published:** 2018-03-23

**Authors:** Yoonyoung Sung, Sookyoung Lee, Meejeong Lee

**Affiliations:** Department of Computer Science and Engineering, Ewha Womans University, Seoul 03760, Korea; syy83@ewhain.net

**Keywords:** Internet of Things, multi-hop cluster, IoT network, scalability, optimization

## Abstract

It is expected that up to 26 billion Internet of Things (IoT) equipped with sensors and wireless communication capabilities will be connected to the Internet by 2020 for various purposes. With a large scale IoT network, having each node connected to the Internet with an individual connection may face serious scalability issues. The scalability problem of the IoT network may be alleviated by grouping the nodes of the IoT network into clusters and having a representative node in each cluster connect to the Internet on behalf of the other nodes in the cluster instead of having a per-node Internet connection and communication. In this paper, we propose a multi-hop clustering mechanism for IoT networks to minimize the number of required Internet connections. Specifically, the objective of proposed mechanism is to select the minimum number of coordinators, which take the role of a representative node for the cluster, i.e., having the Internet connection on behalf of the rest of the nodes in the cluster and to map a partition of the IoT nodes onto the selected set of coordinators to minimize the total distance between the nodes and their respective coordinator under a certain constraint in terms of maximum hop count between the IoT nodes and their respective coordinator. Since this problem can be mapped into a set cover problem which is known as NP-hard, we pursue a heuristic approach to solve the problem and analyze the complexity of the proposed solution. Through a set of experiments with varying parameters, the proposed scheme shows 63–87.3% reduction of the Internet connections depending on the number of the IoT nodes while that of the optimal solution is 65.6–89.9% in a small scale network. Moreover, it is shown that the performance characteristics of the proposed mechanism coincide with expected performance characteristics of the optimal solution in a large-scale network.

## 1. Introduction

The Internet of Things (IoT) relies on the interconnected objects that are able to communicate with each other and collect data about their context [1]. The IoT devices are assumed to be equipped with the network interfaces of low-power short-range wireless protocols, such as Bluetooth 4.0 and Zigbee, so that communications among themselves are possible. Furthermore, recent advances in the low-power long-range communication technologies, such as Bluetooth 5.0 and LTE-M, will facilitate even wider area communications for the IoT devices. The Gartner group predicts that up to 26 billion of things equipped with sensors and wireless communication capabilities will be connected to the Internet by 2020 for various purposes [2].

For application service such as smart city, factory automation or intelligent building management, the IoT devices placed over a particular area may collect and transmit the sensed or their context data to the servers in the Internet in a periodic manner or in real-time. The size of data may vary from the small ones such as temperature or humidity to the relatively larger ones, such as images. For a large scale IoT network, having each IoT device connected to the server with an individual connection may result in the excessive requirement of Internet connections, as well as the waste of wireless resources of an IoT network. Conservation of the Internet and the wireless network resources may be possible by grouping the devices of an IoT network into clusters and having a representative device collects the data from the rest of the devices in each cluster and communicates with the server on behalf of the other devices instead of having a per-device Internet connection and communication [3,4,5]. Hereinafter, let us call those representative IoT devices responsible for the data transmission to the Internet servers as the coordinators and the entire set of IoT devices as nodes. By configuring the IoT networks in this way, the Internet connection is required for the coordinators only instead of the entire nodes in the IoT network. To minimize the number of Internet connections, the clusters need to be configured as large as possible. As the size of the cluster becomes larger, though, the data transmission delay, as well as the wireless resource consumption increases, and congestion may occur, especially near the coordinators. Since it may lead to the degradation of quality of application services, the clusters for IoT networks need to be configured to cover as many nodes as the wireless capacity of IoT network allows.

Clustering of a network into groups has already been extensively studied in the context of sensing data transmissions in the sensor networks. The sensors are equipped with limited energy resources, but are required to operate without recharging or replacing batteries for extended periods of time. Clustering techniques for sensor networks, therefore, have been introduced to support the scalability with energy efficient communication between sensors and eventually to prolong the life time of a network [4]. Note, differing from the typical sensors, the nodes of IoT networks are expected to be the appliances which are equipped with continuous supply of power to perform some intrinsic functionality other than mere sensing. For the IoT networks, therefore, minimizing the number of Internet connections, instead of the energy efficient communications, is the critical issue for the conservation of network management cost.

In this paper, therefore, we propose a clustering mechanism for the IoT networks that have a continuous supply of power. Especially, we assume the leverage of low-power wireless communications, which are supposed to be commonly available at the IoT devices, for the communication between the nodes and their coordinators for the flexible and scalable formation of clusters. Extending the hop counts between a coordinator and its member nodes leads to the increase of delay in the wireless network. The proposed clustering mechanism, hence, tries to minimize the number of required Internet connections while avoiding the excessive delay in the wireless network. Specifically, the objective of proposed mechanism is to select the minimum number of coordinators and to map a partition of IoT nodes onto the selected set of coordinators under a given constraint of maximum hop count between the IoT nodes and their respective coordinators. This problem can be mapped into a set cover problem which is known as NP-hard [6] and we, thus, pursue heuristics.

The proposed mechanism is particularly useful for the IoT network deployed in a very large area, like a city, where a large volume of the smart nodes are monitored or maintained outdoors, such as street lights, traffic lights, noise/pollution/traffic monitoring devices, etc. [7]. Since the smart nodes are sparsely deployed outdoor, they may be out of range of the coverage of Wi-Fi access points located in the city. Therefore, the nodes require a cellular connection for accessing their common server managed by the city. Under such a scenario, using a separate cellular link from each node to the server would result in a high price of using the cellular network as well as a very large amount of overhead for the server. Therefore, it is essential to reduce a count of the selected smart nodes that opens a cellular connection to the server by forming multi-hop clusters in which low-power short-distance radio communication, such as Bluetooth and Zigbee, is used.

This paper is organized as follows: Section 2 summarizes the clustering approaches for wireless sensor networks and explains their inappropriate aspects for IoT environments. In Section 3, the proposed mechanism is explained and its complexity is analyzed. The performance of the proposed mechanism is discussed in Section 4 in two experimental settings. We finally conclude in Section 5.

## 2. Related Works

In sensor network clustering, sensors are grouped into clusters and the representative sensor of a cluster, commonly called as the “cluster head (CH)”, gathers and transmits the sensing data of the other sensors in the cluster to the base station (BS) which connects the sensor network to the outside networks. Typically, the energy consumption of sensors is minimized by having them only communicate with either the nearest neighboring sensor or its CH, which is within one hop distance. The energy consumption of CHs is relatively higher than the other sensors since they have to communicate with the BS which is farther away. Eventually the sensors take turns to undertake the role of CH to balance the energy consumption level among themselves and prolong the lifetime of sensor network.

The clustering mechanisms of sensor networks can be largely classified into the Voronoi-based and the non-Vornoi-based ones [4]. The Voronoi-based mechanisms first select a set of CHs and then have each sensor connected to the nearest CH so that the sensors may communicate with their CH with the minimum energy consumption [8,9,10,11,12,13,14,15]. In the Energy Potential Low-Energy Adaptive Clustering Hierarchy (EP-LEACH) algorithm [9], the CHs are selected among sensors based on the sensor node’s energy harvesting capability. If the energy level of a selected CH falls behind a certain threshold, CH replacement occurs to extend the lifetime of network. The clusters are formed by having each sensor assigned to the closest one-hop away CH and thereby, the energy consumption of sensors is minimized. The Voronoi-based mechanisms are, therefore, not applicable for the objective of configuring clusters as large as possible to minimize the number of Internet connections.

The non-Voronoi-based approach is further classified into the chain-based and the spectrum-based mechanisms [4]. Differing from the Voronoi-based mechanisms, chain-based mechanisms form multi-hop clusters, i.e., the sensors are one or more hops away from their CH. In the chain-based mechanisms, the entire network is formed into chain(s) of sensors having a CH for each chain and the data is transmitted in one direction only following through the chain to be delivered to the CH [16,17,18,19,20,21,22,23,24,25]. The chain is configured so that, for each sensor, the next sensor on the chain is the nearest sensor among the neighboring sensors that are nearer to the BS than itself. Eventually, the sensor(s) that receive data from more than one direction become the CH(s) and the rest of the sensors transmit/receive data to/from a fixed direction only following the chain. The enhanced Power-Efficient GAthering in Sensor Information Systems (Big Sky, MT, USA) (E-PEGASIS) [16] is the chain-based mechanism which opts to reduce the data redundancy to BS. E-PEGASIS finds a dominating set (DS) that includes a subset of deployed nodes to be activated and then the near optimal chain is formed out of DS nodes by using an ant colony optimization. Finally, a chain leader is selected based on residual energy and proximity to BS and then the overhead to BS can be reduced. Meanwhile, the Concentric Clustering Scheme (CCS) [17] proposes a mechanism to configure the network into multiple chains. In CCS, a sensor network is divided into multiple clusters based on the distance from the BS and a chain is formed within each cluster. In the chain-based mechanisms, the energy consumption of sensors can be minimized since a sensor transmits data to the nearest neighboring sensor. The chain-based mechanisms may form long chains and thereby enable to configure large clusters to minimize the number of necessary Internet connections. However, unnecessary detouring in the data delivery paths as well as the extensive delay from sensors to the CH may occur and, thus, it is not applicable to the clustering of IoT networks that have a delay constraint.

In the spectrum-based mechanisms, the sensors are partitioned into fan-shaped sectors centered at the BS and each sector is then divided into multiple cells according to the distance from BS [26,27,28,29]. A cell corresponds to a cluster and a CH is selected for each cell. Due to the characteristic of a sector, the size of a cell which is farther away from the BS is larger than that of the ones nearer to the BS. As a consequence, the farther the cell is from the BS, there exist more sensors in the cell. Since the data transmission requires the higher energy consumption as the distance from the BS becomes farther, the spectrum-based mechanisms try to make cells have even longevity regardless of their distance from the BS by having the farther cells contain more sensors, i.e., more candidates of CH. Since the energy consumption for data transmission is not a prime concern in the clustering of IoT networks that have continuous supply of power, spectrum-based clustering mechanisms are not the optimal solutions for IoT network clustering purposes.

## 3. Multi-Hop Clustering for IoT Networks

It is assumed that the IoT network is composed of a set of IoT nodes, hereafter denoted as $N=\left\{n_{1},\;n_{2},\;\ldots ,n_{j},\ldots \;\right\}$, and all of the nodes are capable of taking the role of a coordinator. With the proposed mechanism, each IoT node $n_{j}$ transfers its data to its selected coordinator instead of transmitting the data directly to an Internet server so that the number of Internet connections required for the IoT network, which is equivalent to the number of the coordinators can be saved. The objective of the proposed mechanism is to find the smallest set C of the selected coordinators $c_{i},\;i=1,\;2,\;\ldots ,k$ for IoT network with N, while satisfying the maximum hop count constraint H between a node $n_{j}$ and its coordinator $c_{i}$. Furthermore, we opt to minimize a sum of hop counts between a coordinator $c_{i}$ and its member nodes, i.e., $\forall n_{j}\in M_{i}$ while minimizing a total count of coordinators, i.e., |C|, where $M_{i}$ is a set of the member nodes which belong to a coordinator $c_{i}$. The objective of the proposed mechanism is then formulated as follows:

$$\mathrm{Minimize}\;\left|C\right|,\;C=\left\{c_{i}\;|\;i=1,\;2,\;\ldots ,k\;\right\},\;C\subset N\;\mathrm{subject}\;\mathrm{to}\;\sum_{\forall c_{i}\;\in \;C}\sum_{\forall n_{j}\;\in \;M_{i}}dist\left(c_{i},n_{j}\right)\;is\;minimized,$$

where

$$M_{i\;}=\left\{\forall n_{j}\in N|\;dist\left(c_{i},n_{j}\right)\leq H,\;c_{i}\in C\right\},\;M_{i}\cap M_{j}=\emptyset \;,\forall i\neq j\;,\;\overset{k}{\underset{i=1}{⋃}}M_{i}=N,\;$$

and dist(a, b)= a hop count in the shortest path between a and b.

In Section 3.1, the proposed mechanism is explained in detail and its complexity is analyzed in Section 3.2.

### 3.1. The Proposed Mechanism

The proposed mechanism is comprised of two steps: (1) trying to compute the smallest set of coordinators which covers all IoT nodes in the network N within a maximum hop count constraint H by repeatedly selecting a node (coordinator) ∈N which can reach the largest number of member nodes within H in a greedy manner and then (2) optimizing a total count of the selected coordinators during the first step by rearranging the mapping of member nodes to the coordinator.

(1) Computing the Smallest Set C of Coordinators Using Greedy Heuristic

The elements of coordinator set, C, are determined in a greedy manner by selecting the node from which the largest number of nodes are reachable within the maximum hop count constraint H. C starts with an empty set, and among the nodes that are not in C yet, the one that may reach out to the largest number of nodes that are not bound to any of the coordinators in C yet is selected as the next coordinator to be included in C. Whenever a node is selected as the coordinator, all of those nodes reachable from that selected coordinator are bound to that coordinator as its member nodes so that they are no longer considered for the selection of next coordinator. This process is repeated until every node is bound to one of the coordinators in C.

In order to apply the processes explained above, the information about the distance among nodes is necessary. Dijkstra’s Shortest Path First (SPF) algorithm is assumed to be used to determine the distance among nodes in the IoT network.

The pseudo-code of the first step of the algorithm is presented in Algorithm 1. First, set $B_{i}$, which is the set of nodes that are reachable from node $n_{i}$ within H, is computed for every node in N (line 1–8). The coordinators are, then, selected one by one by the iteration of while loop from line 10 to 23. At each iteration of the while loop, crd, which is the next coordinator to be included in the coordinator set C is determined by the for loop from line 12 to 17. That is, the node whose $\left|B_{i}\right|$ is the largest among the nodes in $N_{u}$ is determined as crd by the for loop from line 12 to 17. Note $N_{u}$ is the set of nodes that have not been assigned to any coordinator yet and it is initialized with the entire set of nodes N before the while loop starts (line 10). After crd, which is the next coordinator to be included in C, is determined, crd as well as every node reachable from crd (i.e., $B_{crd}$) is removed from $N_{u}$ as well as $B_{i},\;\forall n_{i}\in N_{u}$. The iteration of while loop continues until $N_{u}$ becomes empty (line 10).

**Algorithm 1** the first step of the algorithm.$N=\left\{n_{i}\;|\;i=1,\;2,\;\ldots \right\}$: set of nodes $N_{u}$: nodes that have not been assigned to a coordinator yet $C=\{c_{j}|\;j=1,\;2,\;\ldots ,\;k\}$: set of coordinators H: Constraint on the hop count between a node and its coordinator $B_{i}:$ set of reachable nodes from $n_{i}$ within H
$dist\left(n_{i},\;n_{j}\right)$: distance of shortest path between $n_{i}$ and $n_{j}$ for $n_{i},\;n_{j}\in N$ (Dijsktra’s SPF algorithm is assumed to be used to compute $dist\left(n_{i},\;n_{j}\right)$)   1:  **for** every $n_{i}\in N$
2:     $B_{i}=\emptyset$
3:     **for** every $n_{j}\in N$
4:        **if** ($dist\left(n_{i},n_{j}\right)\leq H$) **then**
5:           $B_{i}=B_{i}\cup^{\text{​}}\left\{n_{j}\right\}$
6:        **end if**
7:     **end for**
8:  **end for**
  9:   $N_{u}=N$, C=∅
10: **while** ($N_{u}!=\emptyset$) **do**
11:    $max\_B_{i}=0$
12:    **for** every $n_{i}\in N_{u}$
13:       **if** ($\left|B_{i}\right|>max\_B_{i}$) **then**
14:          $crd=n_{i}$
15:          $max\_B_{i}=\left|B_{i}\right|$
16:       **end if**
17:    **end for**
18:    $C=C\cup^{\text{​}}\left\{crd\right\}$
19:    $N_{u}=N_{u}-B_{crd}-\left\{crd\right\}$
20:    **for** every $n_{i}\in N_{u}$
21:       $B_{i}=B_{i}-B_{crd}-\left\{crd\right\}$
22:    **end for**
23: **end while**

(2) Optimizing a Total Count of Coordinators in C by Rearranging Member Nodes

Since the previous step tries to find the least set of the selected coordinators by partitioning the member nodes among the selected coordinators, some member nodes may be reachable to multiple coordinators within the maximum hop count constraint H. Therefore, in the second step we reorganize member nodes by changing their membership based on the shortest path. Throughout the second step, we opt to reduce the total number of the chosen coordinators in the first step by switching a coordinator with no member to a member node.

First, let us sequence the coordinators in the order of selection by step (1). In step (1), a node is tentatively assumed to be assigned to the first coordinator that is reachable from itself. Among the coordinators in C, which is determined by step (1), a node may have more than one coordinator reachable within H. A node needs to be reassigned to the best coordinator among the available ones in order to minimize the hop count between a node and its coordinator. Note the delay, as well as the required wireless resource can be decreased by reducing the hop count between a node and the coordinator.

After this rearrangement, some of the coordinators selected by step (1) may turn out to have no member node. For each of those coordinators, it is checked whether it can be served by the other coordinator that has one or more member nodes and if so, the coordinator that has no member node is then eliminated from C to reduce C further. Among the coordinators that can provide the service to the coordinator that has no member node, the nearest one is selected as its coordinator.

The pseudo-code of the second step of the algorithm is presented in Algorithm 2. $M_{i}$, the set of member nodes served by coordinator $c_{i}$, is first initialized to include $c_{i}$ only (lines 1–3). Then, every node $n_{i}$ in N is assigned to the nearest coordinator among the coordinators in C (lines 4–7). Some of the coordinators may left with no member node except for itself after the coordinator assignment is finished at line 8. Each of those coordinators is then removed from C and assigned to the nearest coordinator similar to the other non-coordinator nodes in N if there exist some other coordinators that are reachable within H and have more than one member node (lines 8–14).

**Algorithm 2** The second step of the algorithm$N=\left\{n_{i}\;|\;i=1,\;2,\;\ldots \right\}$: set of nodes $C=\{c_{j}|\;j=1,\;2,\;\ldots ,\;k\}$: set of coordinators $M_{i}$: set of member nodes served by $c_{i}$
$dist\left(n_{i},\;n_{j}\right)$: distance of shortest path between $n_{i}$ and $n_{j}$ for $n_{i},\;n_{j}\in N$ (Dijsktra’s SPF algorithm is assumed to be used to compute $dist\left(n_{i},\;n_{j}\right)$)   1:  **for** every $c_{i}\in C$
2:     $M_{i}=\left\{c_{i}\right\}$
3:  **end for**
  4:  **for** every $n_{i}\in N$
5:     select $c_{j}$ with $dist\left(c_{j},\;n_{i}\right)=\underset{c_{k}\in C}{\min}\left\{dist\left(c_{k},\;n_{i}\right)\right\}$ as the coordinator 6:     $M_{j}=M_{j}\cup^{\text{​}}\left\{n_{i}\right\}$
7:  **end for**
  8:  **for** every $c_{i}\in C$ with $\left|M_{i}\right|=1$
9:     select $c_{j}$ with $dist\left(c_{j},\;c_{i}\right)=\underset{c_{k}\in C\;\&\&\;\left|M_{k}\right|\geq 2\;\&\&dist\left(c_{k},\;c_{i}\right)\leq H}{\min}\left\{dist\left(c_{k},\;c_{i}\right)\right\}$ as the coordinator 10:    **if**
$c_{j}$ is selected **then**
11:       $C=C-\left\{c_{i}\right\}$
12:       $M_{j}=M_{j}\cup^{\text{​}}\left\{c_{i}\right\}$
13:    **end if**
14: **end for**

### 3.2. Complexity Analysis

The complexity of proposed mechanism is analyzed with the following two lemmas and a theorem.

**Lemma** **1.** *Let* N *and* E *be the set of nodes and the edges of IoT network respectively. The time complexity of obtaining the distance among all possible pairs of nodes by applying the Dijkstra’s SPF algorithm is then* O(|N|(|N|log|N|+|E|))*. Furthermore, the time complexity of obtaining* C *is* $O\left({\left|N\right|}^{2}\log\left|N\right|\right)$ *.*

**Proof.**  Implementing with the Fibonacci heap structure, Dijkstra’s SPF algorithm takes O(|N|log|N|+|E|) for the computation of paths from a single source to all nodes [30]. The computation of paths for all possible sources in the network, hence, takes O(|N|(|N|log|N|+|E|)), where $\left|E\right|=O\left({\left|N\right|}^{2}\right)$. Especially when $\left|E\right|={\left|N\right|}^{2},$ the connectivity of given network is a full mesh, and the problem that we are solving is simplified to choosing a single coordinator that is located nearest to the center of the network. Thus the time complexity becomes O(|N|) in this case. □

To obtain $B_{i}$, which is the set of nodes reachable from $n_{i}$, for $n_{i}\in N$, within H hops, the distance between $n_{i}$ and all the other nodes in N needs to be checked. The time to compute $B_{i}$ for a certain $n_{i}\in N$ is, therefore, proportional to the number of nodes |N|, and in turn, the time complexity to compute $B_{i}$ for all $n_{i}\in N$ is $O\left({\left|N\right|}^{2}\right)$.

After obtaining $B_{i}$ for all $n_{i}\in N$, the element of coordinator set C is selected from $N_{u}$ one by one, that is, the $n_{i}$ whose $\left|B_{i}\right|$ is $\underset{n_{j}\in N_{u}}{\max}\left\{\left|B_{j}\right|\right\}$ is selected as the next coordinator to be included in C. When node $n_{i}$ is selected as the next coordinator to be included in C, the selected node $n_{i}$, as well as all the nodes in $B_{i}$, are removed from $N_{u}$ and then from every $B_{j}$ for $n_{j}\in N_{u}$ before proceeding to the selection of next coordinator. The selection continues until $N_{u}=\emptyset$.

For each selection of a coordinator, the comparisons among $\left|B_{j}\right|$ for $n_{j}\in N_{u}$ to select the $n_{i}$ whose $\left|B_{i}\right|$ is $\underset{n_{j}\in N_{u}}{\max}\left\{\left|B_{j}\right|\right\}$ occur |N| times at most since $\left|N_{u}\right|$ is at most |N|. Furthermore, the iterations of this selection step occur at most |N| times to complete the selection of coordinators for the set C. The comparisons among $\left|B_{j}\right|$ for $n_{j}\in N_{u}$, hence, occur ${\left|N\right|}^{2}$ at most, meaning the time complexity of $O\left({\left|N\right|}^{2}\right).$

Since a partition of set N is removed from $N_{u}$ at each selection step and the selection step continues until $N_{u}$ becomes empty, the element removal from $N_{u}$ occurs exactly |N| times in total until the set C is completed. Similarly, the element removal occurs |N| times at most for each $B_{j}$ for $n_{j}\in N_{u}$. The removal of a node from $N_{u}$ or $B_{j}$ for $n_{j}\in N_{u}$ requires the lookup of that specific node in $N_{u}$ or $B_{j}$ for $n_{j}\in N_{u}$, and it requires, at most, log|N| comparisons since $\left|N_{u}\right|$ and $\left|B_{j}\right|$ for $n_{j}\in N_{u}$ are |N|, at most. Since all of nodes in $N_{u}$ have to be removed to complete the selection of coordinators, exactly |N| lookups for $N_{u}$ and at most |N| lookups for each of $B_{j}$ for $n_{j}\in N_{u}$, respectively, are necessary. Therefore, at most |N|log|N| comparisons for $N_{u}$ and $B_{j}$ for $n_{j}\in N_{u}$. Therefore, the time complexity to remove all nodes from $N_{u}$ and $B_{j}$ for all $n_{j}\in N_{u}$, that is, the time complexity of completing the selection of coordinators is $O\left({\left|N\right|}^{2}\log\left|N\right|\right)$.

The procedure of computing the coordinator set C proceeds with computing the initial set of $B_{i}$ for all $n_{i}\in N$, and then selecting the coordinators. Note the time complexity required for the former is $O\left({\left|N\right|}^{2}\right)$, and $O({\left|N\right|}^{2}\log\left|N\right|)$ for the latter. Therefore, the time complexity of obtaining C is $O\left({\left|N\right|}^{2}\log\left|N\right|\right).$

**Lemma** **2.** *The time complexity of assigning the best (closest) coordinator among all the available coordinators in* C *for every node in* N *is* $O\left({\left|N\right|}^{2}\right)$*.*

**Proof.**  Finding the best coordinator for each $n_{i}\in N$ requires the comparison of $dist\left(c_{j},\;n_{i}\right)$ for all $c_{j}\in C$. Since |C| is at most |N|, the time complexity of finding the best coordinator for all $n_{i}\in N$ is $O\left({\left|N\right|}^{2}\right)$. By determining the best coordinator for all $n_{i}\in N$, $M_{j}$, the set of member nodes for each coordinator $c_{j}\in C$ is computed. □

To reduce C further, for $c_{i}$ with $\left|M_{i}\right|=1$, that is, for the coordinator with no member node, $dist\left(c_{j},\;c_{i}\right)$ for all $c_{j}\in C$ with $\left|M_{j}\right|\geq 2$ and $dist\left(c_{j},\;c_{i}\right)\leq H$ are compared to determine whether $c_{i}$ can be eliminated from C, and if so, to choose the best coordinator for $c_{i}$. The number of coordinator $c_{i}$ with $\left|M_{i}\right|=1$ and the number of coordinator $c_{j}$ with $\left|M_{j}\right|\geq 2$ and $dist\left(c_{j},\;c_{i}\right)\leq H$ are at most |C|, and in turn |C| is at most |N|. Therefore, the time complexity to choose coordinators for the coordinators that have no member for further reduction of C is also $O\left({\left|N\right|}^{2}\right)$.

**Theorem** **1.** *The time complexity of proposed mechanism is* $O\left({\left|N\right|}^{2}\log\left|N\right|+\left|N\right|\left|E\right|\right)$*.*

**Proof.**  The time complexity of obtaining the coordinator set C and assigning the best coordinator for each node in N are analyzed in Lemmas 1 and 2, respectively. Therefore, the time complexity of the proposed mechanism is $O\left(\left|N\right|\left(\left|N\right|\log\left|N\right|+\left|E\right|\right)+{\left|N\right|}^{2}\log\left|N\right|+{\left|N\right|}^{2}\right)=\;O\left(\left|N\right|\left(\left|N\right|\log\left|N\right|+\left|E\right|\right)\right),\;\mathrm{where}\;\left|E\right|<O\left({\left|N\right|}^{2}\right)$. That is, the time complexity of proposed mechanism is determined by the Dijkstra’s SPF algorithm to determine $dist\left(n_{i},\;n_{j}\right)$ for all $n_{i},\;n_{j}\in N$. □

## 4. Performance Evaluation

The performance of the proposed mechanism is analyzed and compared to that of the brute-force-based optimal solution. The objective of mechanism is to minimize the number of coordinators while satisfying the QoS requirements, which is represented as the number of maximum hop count from a node to its coordinator, so that the number of required Internet connections is minimized with the given QoS constraint. Furthermore, it is desirable for a node to be connected to the nearest coordinator among the available ones to conserve the wireless resource, as well as to minimize the delay. Hence, the performance metrics are the “number of selected coordinators” and the “average hop counts from a node to its coordinator”. The experimental settings and the compared optimal solutions are first explained in Section 4.1, and in Section 4.2 the numerical results are presented.

### 4.1. Experimental Settings

Selecting the optimal and minimum number of coordinators among the |N| nodes while satisfying the maximum hop count constraint is an NP-hard problem and, thus, the size of the network for which the optimal solution is applicable is very limited. As seen in the pseudo-code of Algorithm 3, the optimal approach opts to find the minimum count *k* of coordinators via which all IoT nodes in N are reachable in one hop by trying all possible sets of *k* coordinators ∈N in rounds. In the first round, the algorithm tries *k* is 1 and checks there is a node $n_{x}$ ∈N from which all nodes ∈N are directly accessible. If such a node $n_{x}$ exists, it becomes a coordinator and the algorithm terminates with *k* = 1. Otherwise in the second round, *k* increases by one and the same procedure is repeated for nodes in each set $\in N_{2}^{j},\;\forall j$ which includes all possible 2(=*k*) combinations of nodes ∈N (lines 3–12). The algorithm terminates when nodes in a set $N_{k}^{x}$ cover all nodes in N and returns *k* and a set of the selected coordinators, $N_{k}^{x}$ (line 10). Clearly, this is a very exhaustive and time-consuming procedure and cannot be practically applied for a large network. Nonetheless, running such brute-force procedure for a small setup helps in qualifying the performance of the proposed algorithm relative to the optimal solution. The optimal solution and proposed mechanism are compared for a small size network of 25 m × 25 m area with 20 to 70 randomly distributed nodes across the network area following the uniform random distribution.

Furthermore, the proposed mechanism is experimented with varying values of parameters that are supposed to affect the number of selected coordinators, that is, the transmission range TR, the maximum hop count constraint H, and the node density of the network. A higher transmission range increases the connectivity among nodes and, as a result, the number of nodes that may be covered by a coordinator tends to grow. A larger maximum hop count constraint H also extends the coverage of a coordinator, which may lead to a smaller number of selected coordinators. Higher node density increases the connectivity of the network and lowers the ratio of selected coordinators to the entire set of nodes in the network. The behavior of the proposed mechanism is analyzed to confirm that it conforms to that of the optimal solution. The parameters of the two kinds of experiments explained above are summarized in Table 1.

**Algorithm 3** The optimal solution$N=\left\{n_{i}\;|\;i=1,\;2,\;\ldots \right\}$: set of nodes $N_{u}$: nodes that have not been assigned to a coordinator yet $C=\{c_{j}|\;j=1,\;2,\;\ldots ,\;k\}$: set of coordinators H: Constraint on the hop count between a node and its coordinator $dist\left(n_{i},\;n_{j}\right)$: distance of shortest path between $n_{i}$ and $n_{j}$ for $n_{i},\;n_{j}\in N$ (Dijsktra’s SPF algorithm is assumed to be used to compute $dist\left(n_{i},\;n_{j}\right)$) $B_{i}:$ set of reachable nodes from $n_{i}$ within H = 1 $N_{k}=\{N_{k}^{j}|k=1,\;2,\;\ldots ,\;\left|N\right|,\;j=1,\;2,\;\ldots ,\;\left(\begin{array}{c} \left|N\right|\\ k \end{array}\right)\}$: set of all possible k-combinations of nodes ∈N
  **Computing**
C**, the set of coordinators**
1:  C=∅
2:  **for**
k=1 to |N|
3:     **for**
j=1 to $\left(\begin{array}{c} \left|N\right|\\ k \end{array}\right)$
4:        $N_{u}=N$
5:        **for**
$n_{x},\;\forall x\in N_{k}^{j}$        // $n_{x}$ is an element of *j*^th^
*k*-combination for N
6:           $N_{u}=N_{u}-B_{x}-\left\{n_{x}\right\}$ // removal of all reachable nodes by $n_{x}$ in 1-hop from $N_{u}$
7:       **end for**
8:        **if** ($N_{u}==\emptyset )$
**then**    // if all nodes ∈N are reachable from the selected nodes ∈
$N_{k}^{j}$
9:          $C=N_{k}^{j}$  // then the nodes in $N_{k}^{j}$ become a coordinator. 10:          return (k, C) 11:       **end if**
12:    **end for**
13: **end for**

### 4.2. Numerical Results

For the two kinds of experimental settings explained in Section 4.1, the number of selected coordinators and the average hop counts from a node to its coordinator are measured. Every result represents the average of 10 measurements with varying node distributions generated according to the uniform random distribution.

#### 4.2.1. Experiments with Small-Scale Networks

**Figure 1:**
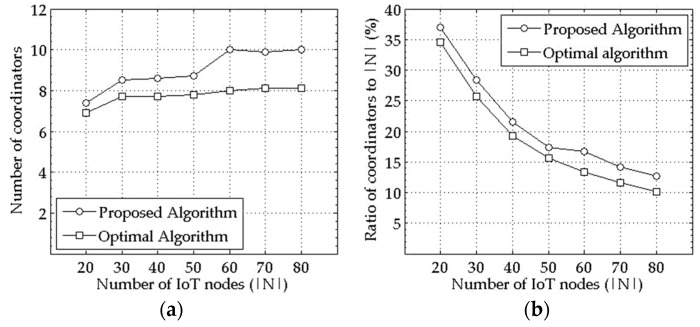
Number (**a**) and ratio (**b**) of selected coordinators in terms of |N|.

Figure 1a shows the average number of selected coordinators for varying the number of nodes in the network with the experimental setting (1) of Table 1 and 0.5 to 2.0 more coordinators are selected by the proposed mechanism than by the optimal solution on the average. It shows, furthermore, the number of selected coordinators converges to a certain value instead of continuing to increase for a given geographical area in the proposed mechanism, as well as in the optimal solution. In other words, not only in the optimal solution but also in the proposed mechanism, it is shown that relatively fewer coordinators to the entire nodes can provide the Internet connection to all IoT nodes in a given network size as the node density increases, as seen in Figure 1b, which shows a ratio of the selected coordinators to all of the nodes in the network.

#### 4.2.2. Experiments with Large-Scale Networks with Varying Parameters

With the proposed mechanism, the number of selected coordinators and the average distance from a node to its corresponding coordinator are obtained for varying transmission range TR, the maximum hop count constraint H, and number of nodes in the network. The effects of TR, H, and the node density to the number of coordinators required by the optimal solution are expected to be as follows:

(1) With the larger TR, the degree of node connectivity becomes higher, which leads to the increase of number of nodes that can be connected to a single coordinator. As a result the number of required coordinators for a network declines.
(2) For a given TR value, the larger maximum hop count constraint allows more nodes to be covered by a coordinator and also leads to a smaller number of coordinators in the network.
(3) As the node density increases, the degree of connectivity among nodes becomes higher and, as a result, the ratio of selected coordinators to the entire nodes becomes lower.

**Figure 2:**
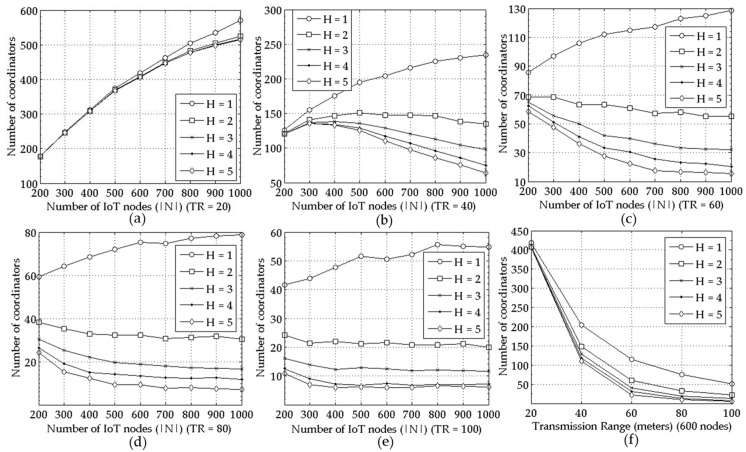
Performance comparison for various *H* values = {1, 2, 3, 4, 5} measured as the number of coordinators under varying |*N*|; for (**a**–**e**) *TR* = 20, 40, …, 100, respectively, and for (**f**) the number of coordinators in terms of various *TR*s with |*N*| = 600.

**Figure 3:**
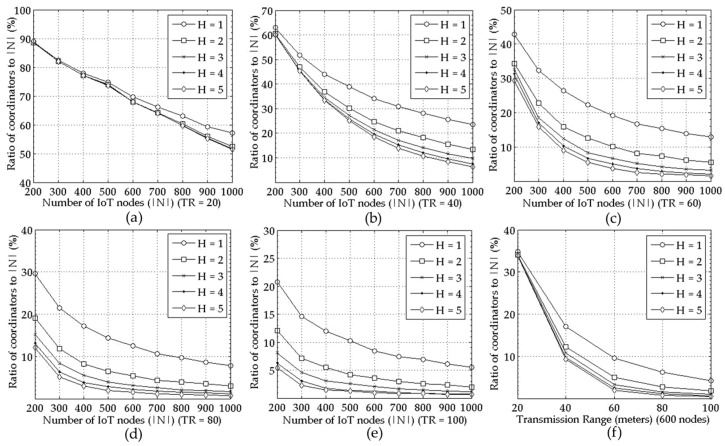
Performance comparison for various *H* values = {1, 2, 3, 4, 5} measured as ratio of coordinators to the entire nodes under varying |*N*|; for (**a**–**e**) *TR* = 20, 40, …, 100, respectively, and for (**f**) the ratio of coordinators to the entire nodes in terms of various *TR*s with |*N*| = 600.

The results obtained from proposed mechanism well align with the above expectations on the optimal solution as shown in Figure 2 and Figure 3. For the transmission range TR of 20, 40, 60, 80, and 100, the number of selected coordinators for increasing node density are shown in Figure 2a–e, respectively. For each of the transmission range values, a maximum hop constraint of 1 to 5 is applied. In Figure 2f, it is shown that when the number of entire nodes is 600, the number of selected coordinators is smaller for larger TR with a given H, and vice versa.

**Figure 4:**
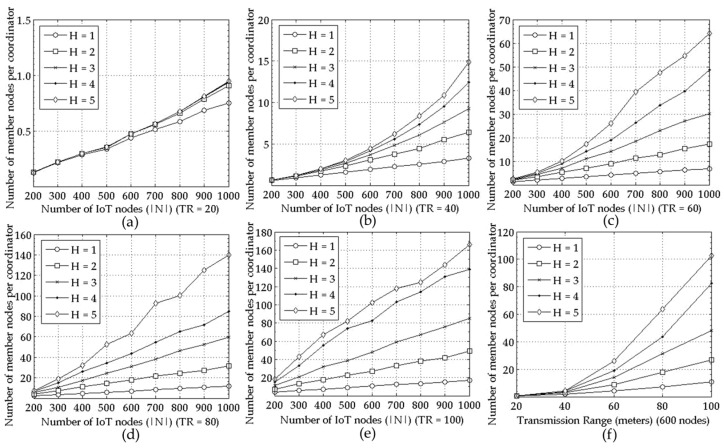
Performance comparison for various *H* values = {1, 2, 3, 4, 5} measured as average number of member nodes for a coordinator under varying |*N*|; for (**a**–**e**) *TR* = 20, 40, …, 100, respectively, and for (**f**) average number of member nodes for a coordinator in terms of various *TR*s with |*N*|=600.

The ratio of coordinators to the entire nodes becomes smaller as the node density increases for all TR and H values in Figure 3. Moreover, Figure 2a–e and Figure 3a–e show that the performance of the proposed mechanism in a large scale network correspond to the one in a small scale network seen in Figure 1a,b respectively. For example, the number of the selected coordinators increases as the number of the IoT nodes, i.e., |*N*| grows regardless of TR as seen in Figure 1a and Figure 2a–e for *H* = 1. Furthermore, it is smaller for larger TR and H. The reason is shown in Figure 4, that is, the average number of member nodes connected to a coordinator increases as the node density, TR, or H increases.

**Figure 5:**
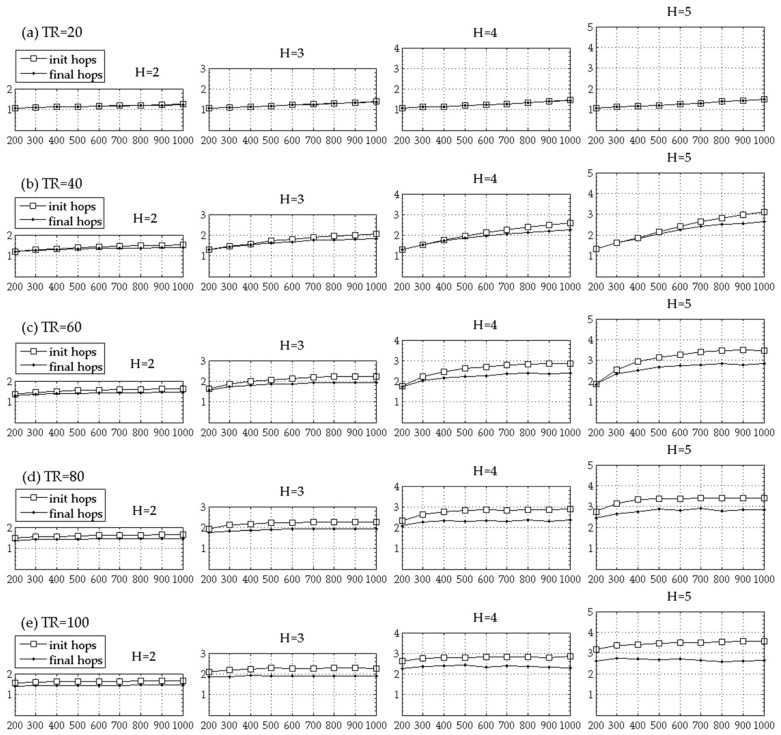
Average hop counts before and after the coordinator reassignment step (*x* axis: number of IoT nodes (|N|), *y* axis: number of hops).

To check the effectiveness of “the coordinator reassignment step” of the proposed mechanism, the hop count from a node to its coordinator is measured before and after the coordinator reassignment step and the average values are compared in Figure 5. If the TR value is small and/or the node density is low, the connectivity among nodes becomes poor and it is more likely that a node connected to a coordinator to which the hop count is less than the maximum hop count constraint. Therefore, the difference between the maximum hop count constraint and the average hop count between nodes and their coordinators is larger for a smaller TR value and for a lower node density at a given *TR* value in Figure 5.

In Figure 5, the difference between the average hop count values before and after the coordinator reassignment step becomes larger for larger H values and as the node density increases and/or the TR becomes larger. This is because more candidate coordinators are available within the maximum hop count constraint as the maximum hop count constraint or the degree of connectivity among the network nodes increases. That is, the effect of the coordinator reassignment step becomes greater as H, TR, or the node density becomes larger.

## 5. Conclusions

We proposed a multi-hop clustering mechanism for IoT networks to minimize the number of required Internet connections. Specifically, the proposed mechanism tries to select the minimum number of coordinators, which take the role of a representative node for the cluster, i.e., having the Internet connection on behalf of the rest of the nodes in the cluster and maps a partition of the IoT nodes onto the selected set of coordinators to minimize the total distance between the nodes and their respective coordinator under a given constraint in terms of the maximum hop count between the IoT nodes and their respective coordinator. Since it is an NP-hard problem, we proposed a heuristic mechanism which has the time complexity of $O\left({\left|N\right|}^{2}\log\left|N\right|+\left|N\right|\left|E\right|\right)$, where |N| and |E| are the number of nodes and the number of wireless links in the IoT network, respectively. The proposed mechanism is applied to a set of networks with varying transmission range and number of nodes with different maximum hop count constraint. The results show that the performance characteristics of proposed mechanism well align with the expected performance characteristics of the optimal solution. That is, the number of selected coordinators reduces as the transmission range and the maximum hop count constraint increase and the ratio of selected coordinators to the entire nodes becomes lower as the node density increases. Our future work includes reducing the complexity of the proposed heuristic algorithm which mainly depends on the complexity of Dijkstra’s SPF algorithm used to compute a set of paths between every IoT node and the remaining IoT nodes.

## Figures and Tables

**Table 1:** Parameters for the two kinds of experimental settings.

Parameters	(1) Small Scale Network for the Comparison with the Optimal Solution	(2) Large Scale Network to Analyze the Performance of the Proposed Mechanism under Varying Parameters
Size of network	25 m × 25 m	1000 m × 1000 m
Transmission range TR	6 m	20, 40, 60, 80, 100 m
Max. hop count constraint H	1 hop	1, 2, 3, 4, 5 hops
No. of nodes	20, 30, 40, 50, 60, 70 nodes	200, 300, 400, 500, 600, 700, 800, 900, 1000 nodes
Distribution of nodes	Uniform random	Uniform random
